# Optimisation of recombinant TNFα production in *Escherichia coli* using GFP fusions and flow cytometry

**DOI:** 10.3389/fbioe.2023.1171823

**Published:** 2023-08-02

**Authors:** Nurul Asma Hasliza Zulkifly, Tania Selas Castiñeiras, Tim W. Overton

**Affiliations:** ^1^ School of Chemical Engineering and Institute of Microbiology and Infection, The University of Birmingham, Birmingham, United Kingdom; ^2^ Cobra Biologics, Keele, United Kingdom

**Keywords:** green fluorescent protein, heterologous protein, fermentation development, fed-batch growth, flow cytometry

## Abstract

*Escherichia coli* is commonly used industrially to manufacture recombinant proteins for biopharmaceutical applications, as well as in academic and industrial settings for R&D purposes. Optimisation of recombinant protein production remains problematic as many proteins are difficult to make, and process conditions must be optimised for each individual protein. An approach to accelerate process development is the use of a green fluorescent protein (GFP) fusions, which can be used to rapidly and simply measure the quantity and folding state of the protein of interest. In this study, we used GFP fusions to optimise production of recombinant human protein tumour necrosis factor (rhTNFα) using a T7 expression system. Flow cytometry was used to measure fluorescence and cell viability on a single cell level to determine culture heterogeneity. Fluorescence measurements were found to be comparable to data generated by subcellular fractionation and SDS-PAGE, a far more time-intensive technique. We compared production of rhTNFα-GFP with that of GFP alone to determine the impact of rhTNFα on expression levels. Optimised shakeflask conditions were then transferred to fed-batch high cell density bioreactor cultures. Finally, the expression of GFP from a p*araBAD* expression vector was compared to the T7 system. We highlight the utility of GFP fusions and flow cytometry for rapid process development.

## Introduction

Biopharmaceuticals represent a major part of the pharmaceutical sector, with the majority of the top-selling drug products worldwide being recombinant proteins. Around one-third of recombinant protein biopharmaceutical products are made in the bacterial host *Escherichia coli* (Walsh, 2018). *E. coli* is an advantageous host for relatively simple proteins that do not require post-translational modifications such as glycosylation (Overton, 2014; Sanchez-Garcia et al., 2016); example products include insulin, granulocyte-colony stimulating factor and antibody fragments (Walsh, 2018). Two broad strategies are used to produce recombinant proteins in *E. coli*: synthesis of correctly-folded, soluble proteins in *E. coli* (Sørensen and Mortensen, 2005); and production of insoluble inclusion bodies in *E. coli* followed by isolation and refolding *in vitro* (Singh and Panda, 2005). The latter strategy affords simple purification and high protein yields, but is only suitable for protein products which can be successfully refolded from inclusion bodies. For proteins where this is not the case, correct folding must occur in *E. coli*.

Many recombinant proteins are difficult to make in a correctly-folded form; various strategies have been used to improve folding, including co-expression of chaperones (Martínez-Alonso et al., 2010), which is successful in only some cases. A generic approach to improving yields of correctly-folded protein is stress minimisation, whereby lowered growth temperature and inducer concentration results in lower rates of growth and protein production, often permitting correct folding (Vera et al., 2007; Sevastsyanovich et al., 2009; Wyre and Overton, 2014b). This approach has previously been used with model proteins (Vera et al., 2007; Sevastsyanovich et al., 2009; Wyre and Overton, 2014b; Hsu et al., 2016; Kasli et al., 2019) and biopharmaceutical products (Castiñeiras et al., 2018; Selas Castiñeiras et al., 2018) in flask cultures and optimised high cell density culture.

Flow cytometry (FCM) is a technique that can rapidly analyse optical and fluorescent properties of individual particles such as cells and bacteria (Rieseberg et al., 2001). Within a microbiology context, FCM can be used to determine bacterial size and shape, and fluorescent dyes can be used to measure aspects of viability and physiology (Nebe-Von-Caron et al., 2000). As FCM is a direct measurement technique which does not require subsequent growth of bacteria, it can detect viable non-culturable (VBNC) bacteria, which cannot be measured using growth-based assays such as colony forming unit determination. As such, it is a useful technique in bioprocess monitoring and development. Productivity of recombinant proteins can be determined using green fluorescent protein (GFP) fusions and FCM. Translational fusion of *gfp* at the C-terminal of a recombinant gene of interest permits measurement of fluorescence which correlates to the quantity and folding state of the recombinant protein; if the recombinant protein folds correctly then GFP also tends to fold correctly and becomes fluorescent, whereas recombinant protein misfolding leads to GFP misfolding and low fluorescence (Waldo et al., 1999; Vera et al., 2007). We have previously used FCM to optimise production of a model recombinant protein (*E. coli* CheY, which readily misfolds when generated at high levels) fused to GFP in both lab-scale (Sevastsyanovich et al., 2009) and bioreactor cultures (Wyre and Overton, 2014b), as well as at other stages of the RPP process (Wyre and Overton, 2014a).

In this study, we used flow cytometry to measure production of the recombinant human protein tumour necrosis factor (rhTNFα, referred to henceforth as TNFα). TNFα is commercially manufactured by Boehringer Ingelheim under the trade name Beromun^®^ (its non-proprietary name is tasonermin), as a treatment for soft-tissue sarcoma. It has previously been used as a model protein for development of cytoplasmic recombinant protein production processes in *E. coli* (Hodgson et al., 2006; Castiñeiras et al., 2018). Here, we monitored production of a TNFα-GFP fusion on a single-cell basis using FCM. In parallel cultures, GFP was expressed from the same plasmid without TNFα. This approach permits not only optimisation of growth conditions for the production of TNFα-GFP, but also comparison of production of GFP *versus* TNFα-GFP and thereby determination of the effect of TNFα on expression strength and heterogeneity. Production of GFP using a T7 RNA polymerase-based expression system was compared to a p*araBAD* system. Shakeflask cultures were used to determine culture productivity and physiology at small scale, then cultures were intensified in fed-batch bioreactors. We demonstrate that flow cytometry is a useful and rapid technique for the optimisation of recombinant protein production processes, comparing different expression systems, different recombinant proteins, and different growth methods.

## Materials and methods

### Strains and plasmids

All strains and plasmids were sourced from Cobra Biologics (Keele, UK). *E. coli* strains BL21-T7 (*F*- *ompT lon hsdS*_*B*_ (*r*_*B*_^*-*^ *m*_*B*_^*-*^) *gal dcm araBAD*::*T7RNAP*) and BL21-A (*F* ^*−*^ *ompT gal dcm lon hsdSB* (*rB* ^*−*^ *mB* ^*−*^) [*malB* ^+^]*K-12(λS) Δ(ara)*) were used. Plasmids p2T7-T7T-KanRev_GFPmut2, referred to here as p2T7-GFP, and p2T7-T7T-KanRev_TNFa-GFPmut2, referred to here as p2T7-TNFaGFP, comprise the gene encoding GFPmut2 or TNFα translationally fused to GFPmut2, under the control of the T7 promoter, and confer kanamycin resistance. The two T7 plasmids were used with BL21-T7, which expresses the T7 RNA polymerase when induced with arabinose. Plasmid pLBAD-GFP encodes GFPmut2 under the control of the *E. coli* p^*araBAD*^ promoter (Guzman et al., 1995); this is derived from pLBAD2 (Selas Castiñeiras et al., 2018) with a pMB1 origin and kanamycin resistance.

### Culture conditions

Shakeflask cultures were grown in 100 mL of terrific broth (12 g L^−1^ Tryptone, 24 g L^−1^ Yeast Extract, both Oxoid, Basingstoke, UK, 16.9 mM KH_2_PO_4_ and 71.8 mM K_2_HPO_4_, pH 7) supplemented with 50 μg mL^−1^ Kanamycin in a 500 mL conical flask. Protein production was induced with arabinose at 0.015% or 0.2% (v/v). The inoculum was grown in 10 mL Luria Bertani broth with 50 μg mL^−1^ Kanamycin for 18 h at 30°C.

Bioreactor cultures were based on the protocols developed by (Want et al., 2009; Wyre and Overton, 2014b) with a semi-defined medium and glycerol feed. Cultures were grown in a FerMac 310/60 bioreactor (Electrolab, Tewkesbury, UK) with a 5 L glass vessel equipped with four baffles and 2 Rushton turbines. The vessel was sparged with air (sterilised through a 0.22 μm filter) and DOT was measured using a D150 Oxyprobe (Broadly James). DOT was maintained at 30% by controlling agitation rate from 300 rpm to 800 rpm. The pH was maintained at 6.3 via addition of 10% (v/v) NH_3_ or 5% (v/v) HCl. Growth temperature was 30°C. Batch growth used 1.5 L of semi-defined medium (Wyre and Overton, 2014b) containing 35 g L^−1^ glycerol as a carbon source. The overnight culture for bioreactor growth comprised 35 mL of Luria Bertani broth with 50 μg mL^−1^ Kanamycin in a 250 mL conical flask inoculated with a sweep of cells from an agar plate, grown for 18–21 h at 150 rpm, 25°C. A 5 mL sample was taken for analysis and 30 mL was used to inoculate the bioreactor. A total of 500 mL feed (714 g L^−1^ glycerol, 7.4 g L^−1^ MgSO_4_·7H_2_O, 4 mM arabinose) was added according to an exponential feed rate equation (Strandberg, 1994):

$$F=\left(\frac{1}{S}\right)\times \left(\frac{\mu }{Y_{XS}}+m\right)\times X_{0}\times e^{\mu t}$$

Where: *F* is feed rate into the bioreactor (Lh^−1^); *X*_*0*_ is total biomass in bioreactor at start of feed (g DCW); *μ* is specific growth rate, set at 0.2 h^−1^; t is time (h); *S* is feed glycerol concentration (714 g L^−1^); *Y*_*XS*_ is biomass yield coefficient [0.19 g biomass g glycerol^−1^ (Passarinha et al., 2009)]; and *m* is maintenance coefficient [0.025 g glycerol g biomass^−1^h^−1^ (Babaeipour et al., 2007)]. Feeding started at an OD_650_ of 40–50, when the DOT increased indicating that the glycerol in the batch medium had run out. Recombinant protein production was induced by the arabinose in the feed.

### Analytical methods

Culture optical density was measured at 650 nm using an Evolution 200 spectrophotometer (Thermo Fisher Scientific, UK). Colony forming units were determined by diluting the culture in PBS (8 g L^−1^ NaCl, 0.2 g L^−1^ KCl, 1.15 g L^−1^ Na_2_HPO_4_, 0.2 g L^−1^ NaH_2_PO_4_, pH 7.3; Oxoid), plating onto nutrient agar (Oxoid) and incubating for 24 h at 30°C. Plasmid retention was determined by replica-plating onto nutrient agar supplemented with kanamycin and incubation for 24 h at 30°C*.*

### Flow cytometry

Cultures were analysed using a BD Accuri C6 flow cytometer (BD, Oxford, UK). Propidium iodide (PI; Sigma-Aldrich, Poole, UK) was used to stain dead cells; a 200 μg mL^−1^ working solution was used and added to samples to give a PI concentration of 4 μg mL^-1^. Cells were illuminated with a 488 nm laser and fluorescence measured through 533/30 (GFP) and 670 LP (PI) filters. A total of 25 000 events were recorded per sample at an event rate of 1000—2500 s^−1^ using a forward scatter threshold to eliminate particulate noise. Data were collected and analysed using CFlow (BD, Oxford, UK).

**FIGURE 1:**
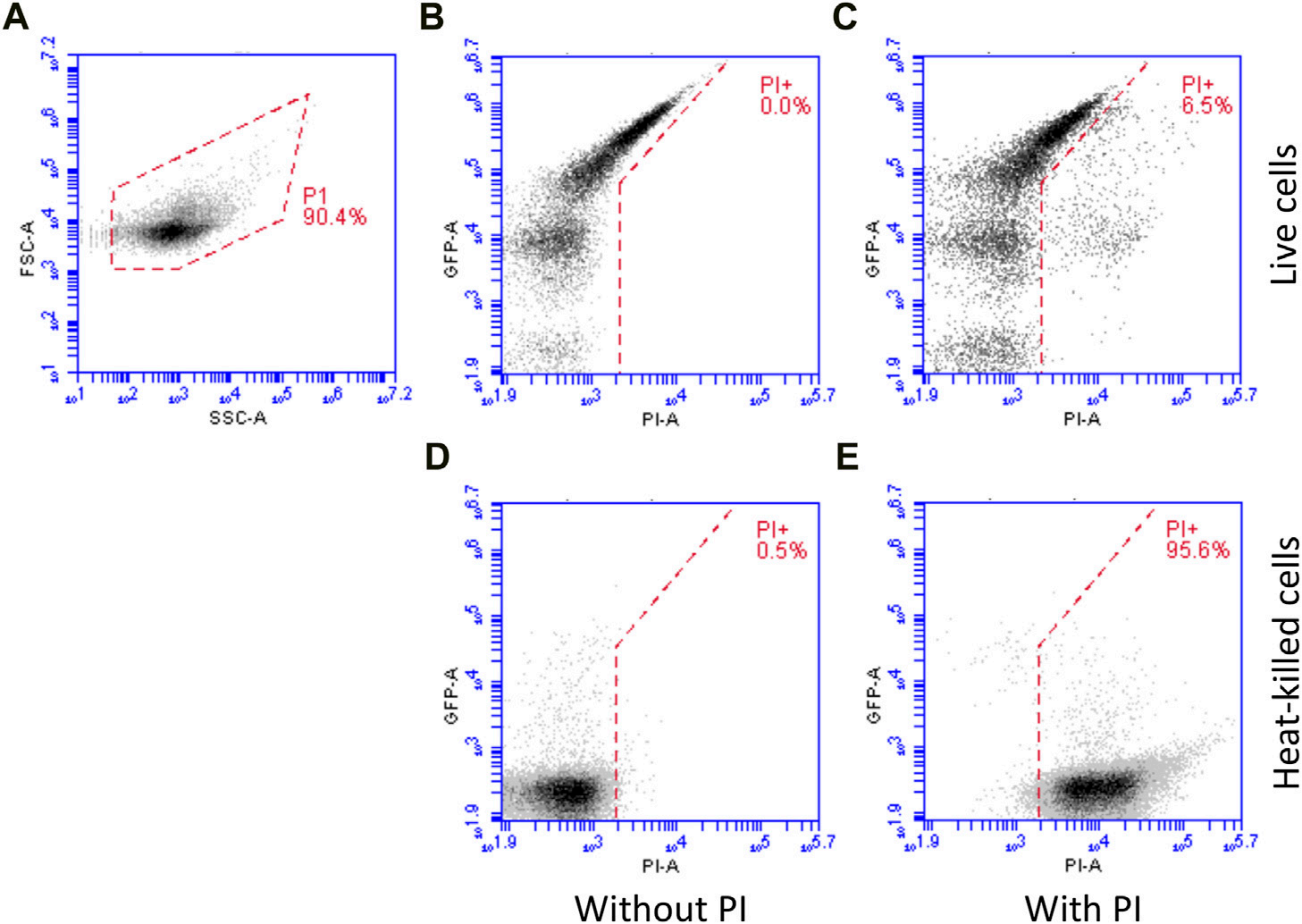
Flow cytometry workflow. For each sample, data were collected using an FSC-H (forward scatter height signal) threshold that eliminated the vast majority of particulate noise and were plotted on an FSC-A *vs*. SSC-A (forward scatter *versus* side scatter) plot and a gate (P1) used to select cells **(A)** which were used for subsequent analysis. For live/dead analysis, samples were stained with PI (which only enters dead cells). Example data is shown: live cells without **(B)** and with **(C)** PI staining, demonstrating little increase in red fluorescence (PI, X axis) and heat-killed cells without **(D)** and with **(E)** PI staining, demonstrating a large increase in red fluorescence.

Flow cytometry data was analysed according to the following workflow (Figure 1), as previously used (Sevastsyanovich et al., 2009; Wyre and Overton, 2014b; Hothersall et al., 2021; Hothersall et al., 2022a; Hothersall et al., 2022b). For each sample, data were collected using an FSC-H (forward scatter height signal) threshold that eliminated the vast majority of particulate noise. The data were plotted on an FSC-A *vs*. SSC-A (forward scatter *versus* side scatter) plot and a gate (P1) used to select cells (Figure 1A). This gate was drawn based on samples with and without cells. Cells within gate P1 were used for subsequent analysis. Mean green fluorescence values and the green fluorescence histograms in Supplementary Figures were derived from samples unstained with PI.

For live/dead analysis, samples were stained with PI (which only enters dead cells) and were gated as above. Cells in gate P1 were plotted on a green fluorescence (GFP) *versus* red fluorescence (PI) plot (Figures 1B, C) and gated according to red fluorescence. Compensation was not used; rather, the line separating PI^+^ from PI^−^ cells was drawn diagonally such that highly green fluorescent cells (where there is some spillover into the red fluorescence channel) were still in the PI^−^ sector of the plot. Figure 1B shows cells without PI and Figure 1C with PI, demonstrating the gating principle. Heat-killed cells (an overnight culture) were used to validate PI staining: Figure 1D shows heat-killed cells without PI staining; whereas Figure 1E shows heat-killed cells stained with PI, resulting in an increase in red fluorescence. Note also that heat killing reduces GFP fluorescence due to denaturation.

### SDS-PAGE

Proteins were analysed by SDS-PAGE. SDS-PAGE gels used for whole cell protein analysis were lab-poured 15% polyacrylamide Tris/Glycine gels prepared as (Wyre and Overton, 2014b). Cell pellets equivalent to 1 mL·OD_650_ were collected by centrifugation (13 000 g, 5 min) and resuspended in 60 μL of sample buffer, boiled for 10 min, and 10 μL of each sample was loaded onto the gel. Gels were run at 110 V for 2 h at room temperature. A molecular weight ladder was run on all gels (5 μL of 0.2 mg mL^−1^ Blue Prestained Protein Standards ladder, New England BioLabs, United States).

To visualise protein bands, gels were stained with SimplyBlue™ Safe Stain (ThermoFisher Scientific, UK), then destained with water, and imaged on a flatbed scanner CanoScan 9000F (Canon, UK) at 4800 dpi resolution. The images were analysed densitometrically using ImageJ software (Schneider et al., 2012). Firstly, images were subjected to background subtraction (default settings) followed by lane description. GFP/TNFα-GFP proteins peaks were defined and the areas under the peak were calculated. To estimate the percentage of protein that was GFP/TNFα-GFP, the corresponding peak intensity was compared to the intensity of the total lane.

### Soluble/insoluble fractionation

Soluble and insoluble proteins were separated by a freeze-thaw method. A volume of culture equivalent to 1 mL·OD_650_ was harvested by centrifugation (13 000 g, 5 min) and the pellet resuspended in 250 μL of pH 8 PBS buffer. Lysozyme (Sigma-Aldrich) was added to 200 μg mL^−1^, followed by 1 μL of Benzonase (Merck Millipore). Samples were placed on ice for 30–45 min before being subjected to 3 freeze-thaw cycles (dry ice/ethanol bath followed by 37°C). Samples were centrifuged (30 min, 9000 g), the soluble fraction (supernatant) removed to a fresh tube, and insoluble fraction (pellet) resuspended in 250 μL of pH 8 PBS. Fractions were separated by SDS-PAGE using Novex WedgeWell Tris-Glycine SDS precast 12% (w/v) polyacrylamide gels (Thermo Fisher). Fractions were mixed with 60 μL of sample buffer, heated (85°C, 2 min) and 10 μL of each sample loaded onto the gel. Following running (200 V, 45 min, room temperature) and staining (as above), gels were scanned and analysed as above; GFP/TNFα-GFP peak areas for soluble and insoluble samples were compared to calculate the solubility.

## Results and discussion

### T7 expression system

Initial experiments used an arabinose-induced T7 expression system, sourced from Cobra Biologics Ltd (Keele, UK). This system is similar to the commonly-used commercial pET/DE3 system (Studier, 1991) except that expression of the chromosomally-encoded T7 RNA polymerase is induced in the BL21-T7 strain by addition of arabinose (Castiñeiras et al., 2018). Two plasmids were used: p2T7-TNFaGFP, where the gene encoding TNFα is expressed under the control of a T7 RNA polymerase-dependent promoter and its C-terminal is translationally fused to GFPmut2 (Cormack et al., 1996); and p2T7-GFP, where the gene encoding TNFα is omitted, so GFPmut2 alone is synthesised. This allowed comparison of the synthesis of GFP *versus* that of TNFα-GFP.

**FIGURE 2:**
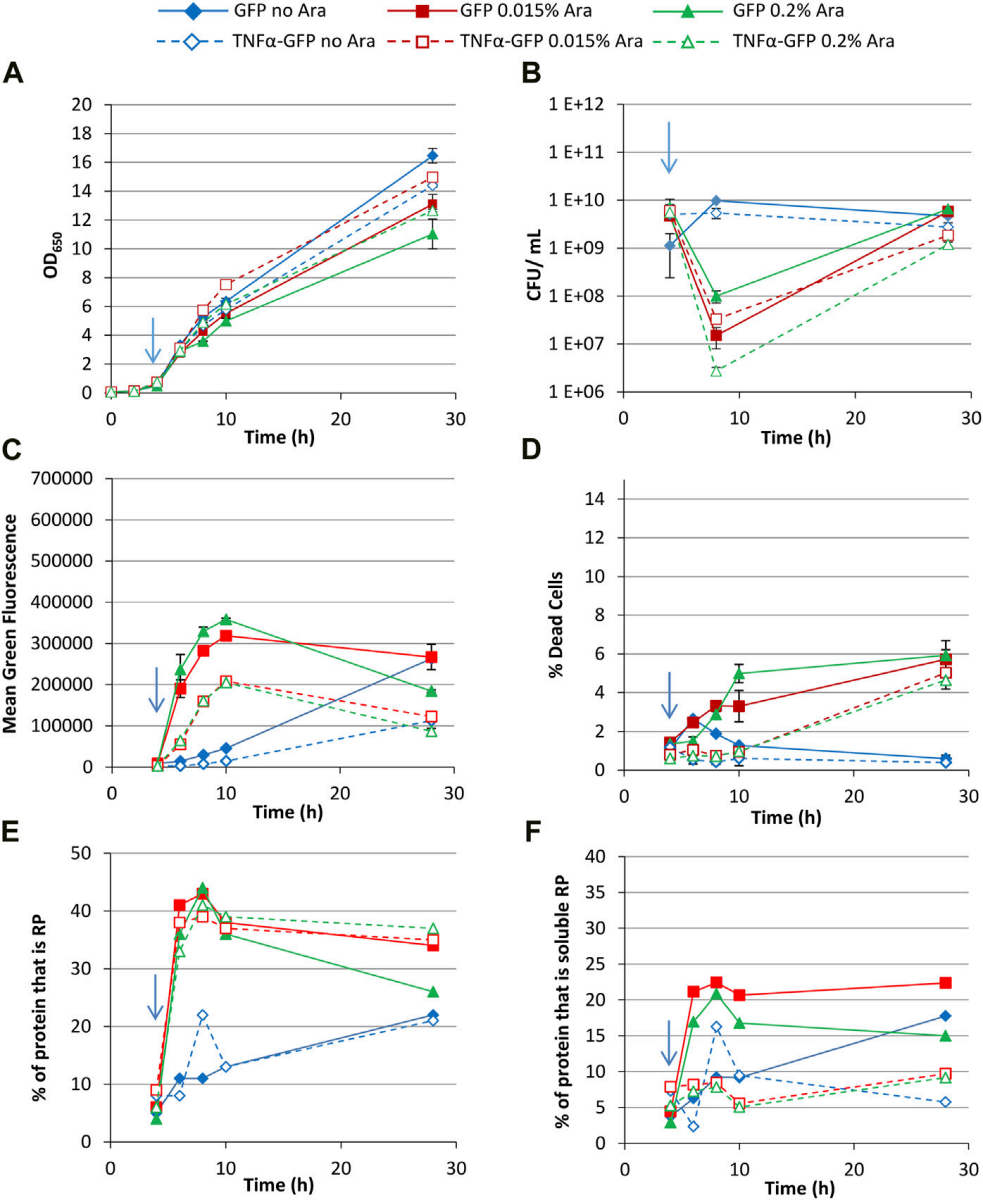
T7 expression system at 30°C with 0.4% glycerol. *E. coli* BL21-T7 cultures carrying either p2T7-TNFaGFP (TNFα-GFP) or p2T7-GFP (GFP) were grown at 30°C in terrific broth with 0.4% glycerol as a carbon source. At an OD_650_ of 0.5 (indicated by an arrow), arabinose (0.015% or 0.2%) was added to four cultures and two were left uninduced. At regular intervals, samples were taken and OD_650_ **(A)** and CFU **(B)** were measured. FCM was used to determine mean green fluorescence **(C)** and percentage of dead cells **(D)** when stained with propidium iodide. Samples were separated by SDS-PAGE and insoluble and soluble proteins separated prior to SDS-PAGE analysis and the percentage of cellular protein, that is, recombinant protein **(E)** and the percentage of cellular protein, that is, soluble recombinant protein **(F)** determined. Data points are mean values of duplicate cultures ± SD.

First, growth conditions were selected that were expected to permit production of soluble recombinant protein; growth at 30°C in Terrific Broth with 0.4% glycerol as a carbon source (which unlike glucose does not repress the expression system). Arabinose (0.015% or 0.2%) was added at an OD_650_ of around 0.5 (after approx. 4 h growth) to induce recombinant protein production, and non-induced controls were included. Induction with high concentrations of arabinose was shown to repress growth (Figure 2A), and culturability decreased immediately after induction as determined by colony forming unit (CFU) assay (Figure 2B), although this increased over time.

Flow cytometry measurement of green fluorescence revealed that cultures synthesising GFP had higher fluorescence than their corresponding TNFα-GFP cultures at all timepoints (Figure 2C). The higher arabinose concentration did not significantly increase fluorescence suggesting that 0.015% arabinose is sufficient to fully activate the promoter. Maximum green fluorescence for induced cultures was after 10 h (6 h post-induction), and similar for both arabinose concentrations, whereas non-induced cultures reached maximum values after 28 h growth due to the leakiness of the promoter. Analysis of the heterogeneity of green fluorescence on a single cell level (Supplementary Figure S1) revealed that non-induced cultures had a small non-productive population (around 2 × 10^2^ fluorescence units, likely plasmid free cells as discussed below) and a much larger single population of GFP^+^ cells at all timepoints, including 4 h (pre-induction), again reflecting the leakiness of the promoter.

Induced cultures showed a similar pattern up to 10 h growth, although at 28 h cultures expressing both GFP and TNFα-GFP comprised multiple populations with different green fluorescence, likely reflecting subpopulations with different RP accumulation and folding state (Wyre and Overton, 2014a). A maximum of 7%–10% of cells were GFP^−^ (around 2 × 10^2^ fluorescence units, representing *E. coli* autofluorescence and the same as untransformed cells, Supplementary Figure S2) indicating that plasmid selection was sufficient to prevent formation of a significant plasmid-free population. The use of kanamycin in this work leads to tighter plasmid selection; comparison with work using plasmids conferring ampicillin resistance reveals far looser selection and much more plasmid loss, e.g., (Sevastsyanovich et al., 2009; Wyre and Overton, 2014b). Staining with propidium iodide (PI) revealed that induced cultures contained more dead cells than uninduced, although less than 6% of cells were dead in induced cultures (Figure 2D). Cultures expressing TNFα-GFP did not have significantly more dead cells than those expressing GFP. For all cultures, the majority of dead cells were GFP^+^.

SDS-PAGE was used to determine the quantity of recombinant protein produced and analysed as the percentage of cellular protein that was the recombinant protein (Figure 2E); up to 10 h growth (6 h post-induction) recombinant protein content was comparable in all induced cultures, irrespective of inducer concentration or whether TNFα-GFP or GFP was being made. At 28 h, cultures synthesising GFP induced with 0.2% arabinose contained less RP than the other three induced conditions. Similarly, uninduced cultures contained comparable proportions of recombinant protein. Fractionation of soluble and insoluble proteins allowed determination of the proportion of protein within the cell that was soluble recombinant protein (Figure 2F); GFP was more soluble than TNFα-GFP, and higher arabinose concentration lowered GFP solubility. Comparison of soluble RP content (Figure 2F) with mean green fluorescence as determined by flow cytometry (Figure 2C) shows broad comparability between the two measurements. The advantage of flow cytometry is its rapidity (typically generating data within 5–10 min) compared to SDS-PAGE which takes hours to generate data. As such flow cytometry can be used as a process analytical tool in real time and results can be used to modify processes on-the-fly. The mean green fluorescence value generated by FCM could be multiplied by the number of cells per unit volume to generate an indication of the quantity of soluble RP in the culture. Overall, the data demonstrates that whereas TNFα-GFP and GFP are produced to similar levels (Figure 2E), their difference in solubility (Figure 2F) leads to their difference in fluorescence (Figure 2C). TNFα-GFP is harder for *E. coli* to synthesise in a functional (fluorescent) form than GFP alone.

### Use of glucose as a carbon source

**FIGURE 3:**
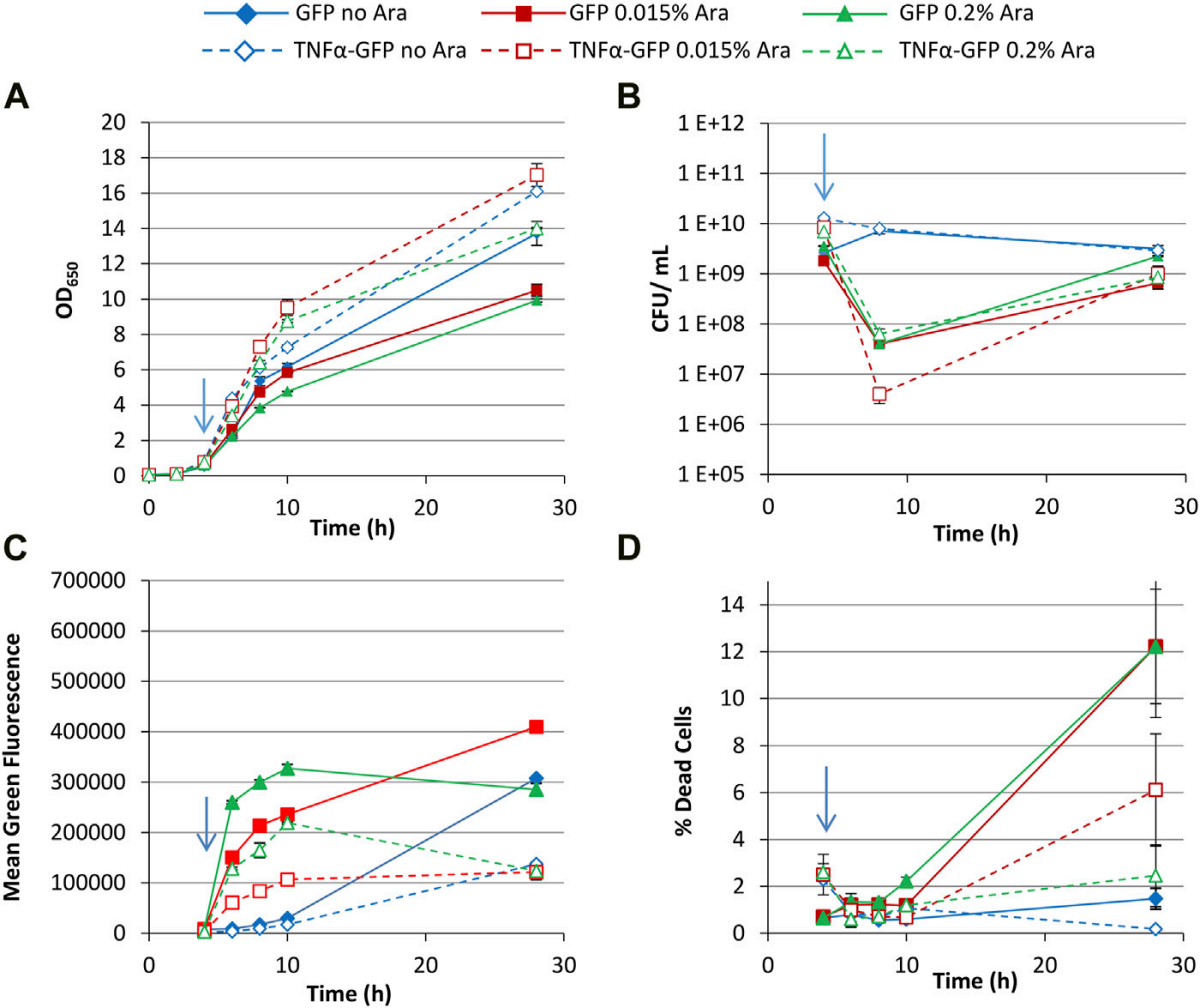
T7 expression system at 30°C with 0.4% glucose. *E. coli* BL21-T7 cultures carrying either p2T7-TNFaGFP (TNFα-GFP) or p2T7-GFP (GFP) were grown at 30°C in terrific broth with 0.4% glucose as a carbon source. At an OD_650_ of 0.5 (indicated by an arrow), arabinose (0.015% or 0.2%) was added to four cultures and two were left uninduced. At regular intervals, samples were taken and OD_650_ **(A)** and CFU **(B)** were measured. FCM was used to determine mean green fluorescence **(C)** and percentage of dead cells **(D)** when stained with propidium iodide. Data points are mean values of duplicate cultures ± SD.

The above experiment was repeated using 0.4% glucose as a carbon source, which represses the arabinose-inducible promoter that regulates T7 RNAP synthesis. Growth of cultures generating GFP was slower than TNFα-GFP cultures (Figure 3A); CFU data was comparable to that of glycerol cultures (Figure 3B), showing a drop then recovery in culturability. Comparison of FCM data revealed that, unlike cultures grown with glycerol (Figure 2C) where different arabinose concentrations did not significantly change the green fluorescence of the bacteria, glucose decreased the production of GFP or TNFα-GFP in cultures induced with the lower (0.015%) concentration of arabinose (Figure 3C). This “dampening” effect has previously been observed, whereby repression of the *araBAD* promoter by glucose and activation by arabinose can be used to “fine tune” expression of recombinant proteins (Castiñeiras et al., 2018). Individual green fluorescence histograms (Supplementary Figure S3) revealed partial induction by 0.015% arabinose, represented by a shoulder on the left of the fluorescence peak that was most distinct at 8 h (2 hours post induction) but still visible at 10 h. No such partial induction was observed with 0.2% arabinose. At 28 h, multiple peaks were observed in all induced cultures, with TNFα-GFP 0.2% arabinose cultures having a significant GFP^−^ peak (∼2 × 10^2^ units) corresponding to non-productive/plasmid-free cells. PI staining revealed that GFP-expressing cultures contained more dead cells than TNFα-GFP cultures (Figure 3D), a probable explanation for the lower OD_650_ values recorded for GFP cultures (Figure 3A).

**FIGURE 4:**
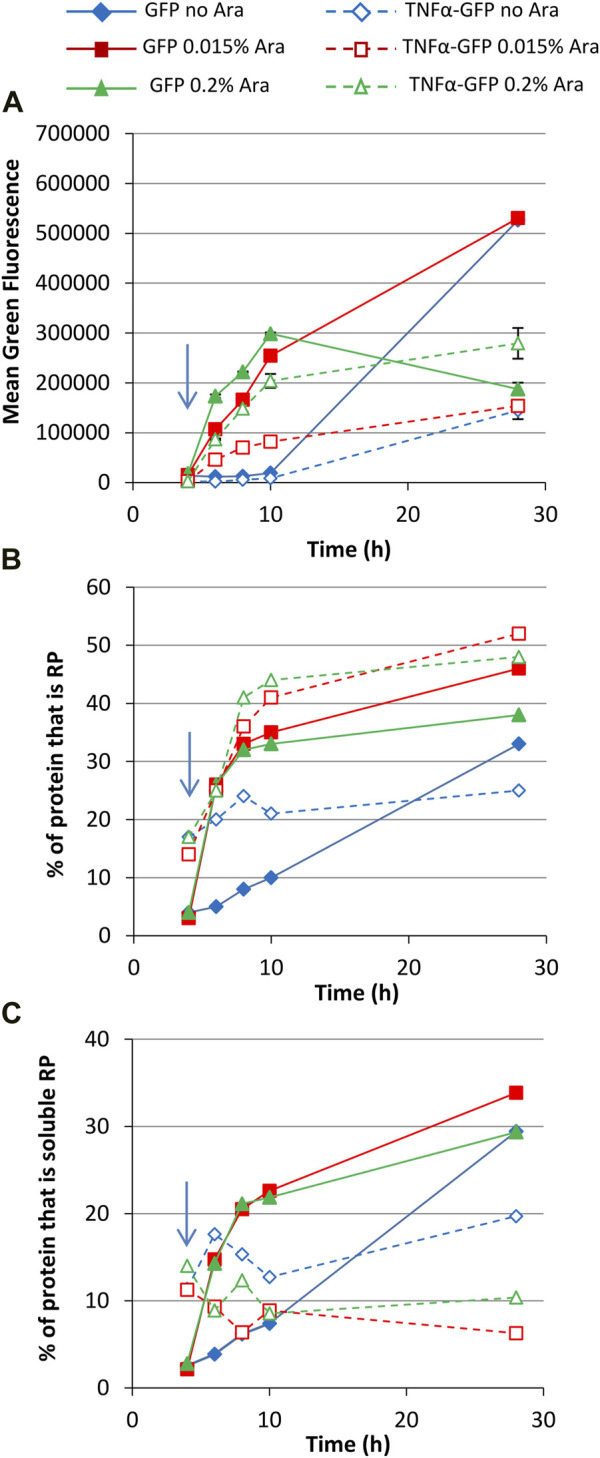
T7 expression system at 30°C with 0.8% glucose. *E. coli* BL21-T7 cultures carrying either p2T7-TNFaGFP (TNFα-GFP) or p2T7-GFP (GFP) were grown at 30°C in terrific broth with 0.8% glucose as a carbon source. At an OD_650_ of 0.5 (indicated by an arrow), arabinose (0.015% or 0.2%) was added to four cultures and two were left uninduced. At regular intervals, samples were taken and FCM was used to determine mean green fluorescence **(A)**. Samples were separated by SDS-PAGE and insoluble and soluble proteins separated prior to SDS-PAGE analysis and the percentage of cellular protein, that is, recombinant protein **(B)** and the percentage of cellular protein, that is, soluble recombinant protein **(C)** determined. Data points are mean values of duplicate cultures ± SD.

Increasing the glucose concentration to 0.8% gave very similar growth profiles to the 0.4% glucose cultures (Supplementary Figure S2). Mean green fluorescence over time was similar to 0.4% glucose cultures (Figure 4A) although fluorescence values for some cultures were higher at 28 h, including uninduced and 0.015% arabinose GFP and 0.2% arabinose TNFα-GFP cultures. This might reflect the higher carbon source concentration leading to less metabolic burden in these cultures. Although biomass concentrations in 0.8% glucose cultures were comparable to 0.4% glycerol cultures, more of the carbon source could have been converted to recombinant protein.

Green fluorescence histograms revealed more pronounced partial induction of TNFα-GFP expression at an arabinose concentration of 0.015% (revealed by the large left shoulder on green fluorescence peaks) and fewer GFP^−^ cells than in the 0.4% glucose cultures (Supplementary Figures S3D, E). SDS-PAGE analysis (Figures 4B, C) was comparable to 0.4% glycerol cultures in that the amount of GFP or TNFα-GFP made was similar for 0.015% and 0.2% arabinose concentration, although TNFα-GFP production was higher in the presence of 0.8% glucose. Flow cytometry green fluorescence data were also broadly reflective of recombinant protein solubility.

### Increasing growth temperature

**FIGURE 5:**
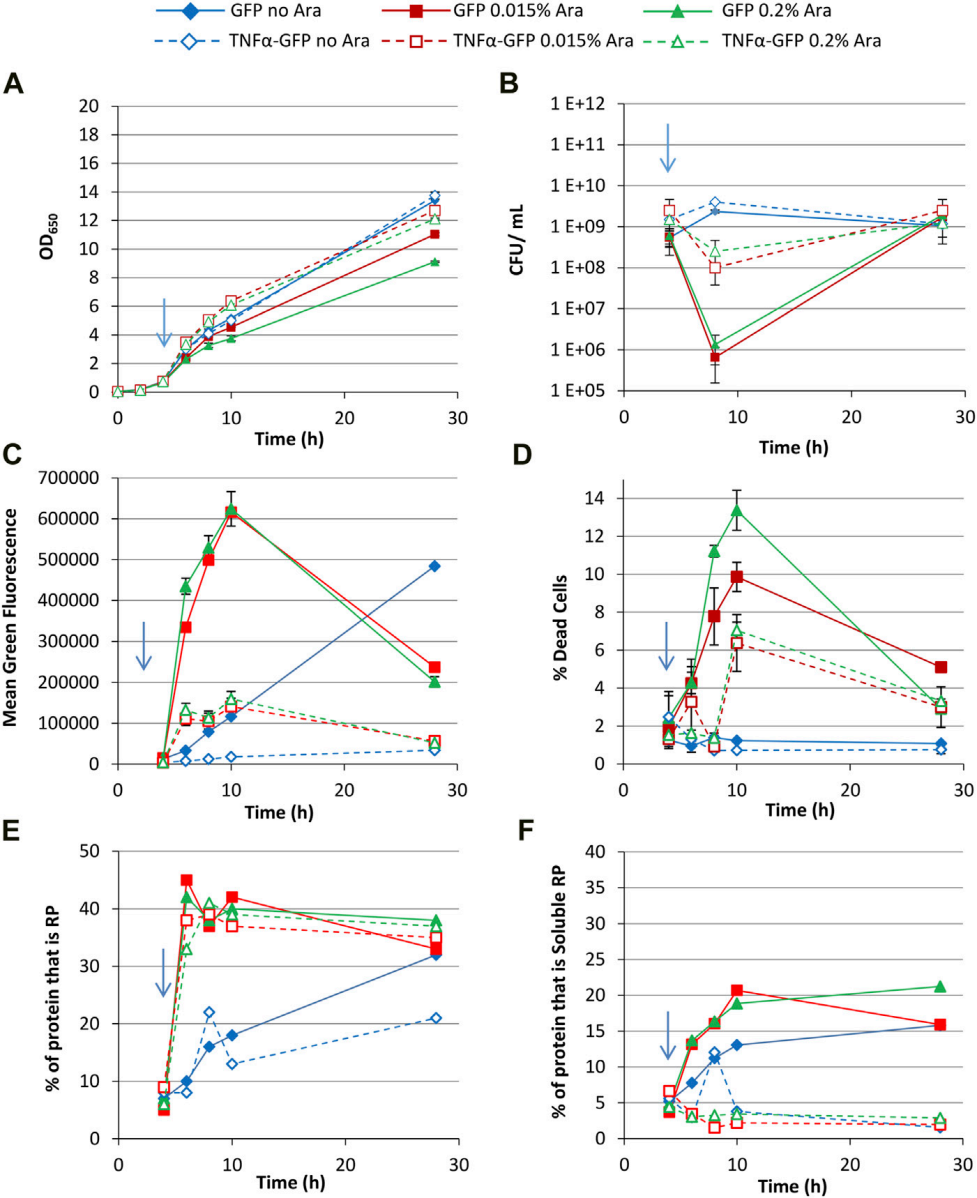
T7 expression system at 37°C with 0.4% glycerol. *E. coli* BL21-T7 cultures carrying either p2T7-TNFaGFP (TNFα-GFP) or p2T7-GFP (GFP) were grown at 37°C in terrific broth with 0.4% glycerol as a carbon source. At an OD_650_ of 0.5 (indicated by an arrow), arabinose (0.015% or 0.2%) was added to four cultures and two were left uninduced. At regular intervals, samples were taken and OD_650_ **(A)** and CFU **(B)** were measured. FCM was used to determine mean green fluorescence **(C)** and percentage of dead cells **(D)** when stained with propidium iodide. Samples were separated by SDS-PAGE and insoluble and soluble proteins separated prior to SDS-PAGE analysis and the percentage of cellular protein, that is, recombinant protein **(E)** and the percentage of cellular protein, that is, soluble recombinant protein **(F)** determined. Data points are mean values of duplicate cultures ± SD.

Increasing the growth temperature to 37°C with 0.4% glycerol as a carbon source resulted in slower growth than at 30°C and a lower final OD_650_ being reached by induced cultures (Figure 5A), presumably due to the increased physiological stress imposed by RPP at 37°C (Vera et al., 2007; Sevastsyanovich et al., 2009; Wyre and Overton, 2014b). This is reflected by a larger drop in CFU (Figure 5B) and increase in dead PI^+^ cells (Figure 5D) than at 30°C. Non-induced cultures grown at 37°C achieved a slightly lower final OD_650_ than at 30°C but there was no significant effect on dead cells or culturability, presumably because the slow accumulation of recombinant protein in these cultures generated less stress than the rapid production in induced cultures. Green fluorescence of GFP-producing cells was higher than at 30°C, reflecting increased GFP production at 37°C (Figure 5C), both arabinose concentrations leading to similar GFP production. TNFα-GFP fluorescence at 37°C was lower than at 30°C for induced and uninduced cultures. Single cell analysis of green fluorescence is similar to 30°C cultures; a single GFP^+^ population following induction, multiple GFP^+^ populations at 28 h in induced cultures, and a very low proportion of GFP^−^ cells (Supplementary Figure S5).

Fractionation and SDS-PAGE revealed that around 30% of GFP was soluble 2 h after induction, but the proportion of soluble GFP increased with time to around 50% (Figures 5B, C). In comparison, in 30°C cultures around 50% of GFP was soluble from 2 h after induction (Figures 2E, F), suggesting better folding at the lower growth temperature. Nonetheless, GFP could still fold and become fluorescent at 37°C, and cultures grown at 37°C had higher fluorescence than at 30°C. Arabinose concentration did not alter GFP solubility at 37°C to a great extent. On the other hand, SDS-PAGE revealed that TNFα-GFP solubility was far lower at 37°C than 30°C. This, along with the lower fluorescence observed by flow cytometry, corroborates previous work showing that TNFα solubility was far higher in *E. coli* at 30°C than 37°C (Castiñeiras et al., 2018). Taken together, GFP appears to be far more tolerant to production at 37°C than TNFα-GFP.

### Use of the *araBAD* promoter

As well as using a T7 polymerase-based expression system, a system based on the *E. coli* arabinose promoter was tested for production of GFP. This system was sourced from Cobra Biologics (Selas Castiñeiras et al., 2018) and is comparable to the commercial pBAD system (Guzman et al., 1995; Invitrogen, 2010). Cultures were grown in the four conditions tested above for the T7 system. Growth of *araBAD* promoter cultures were slower with 0.2% arabinose but overall growth curves were comparable (Supplementary Figure S6). There was no drop in CFU following induction and there were low proportions of dead cells as measured by PI staining. This indicates that the *araBAD* promoter system is better-tolerated and has a far less negative impact on bacterial physiology than the T7 system.

**FIGURE 6:**
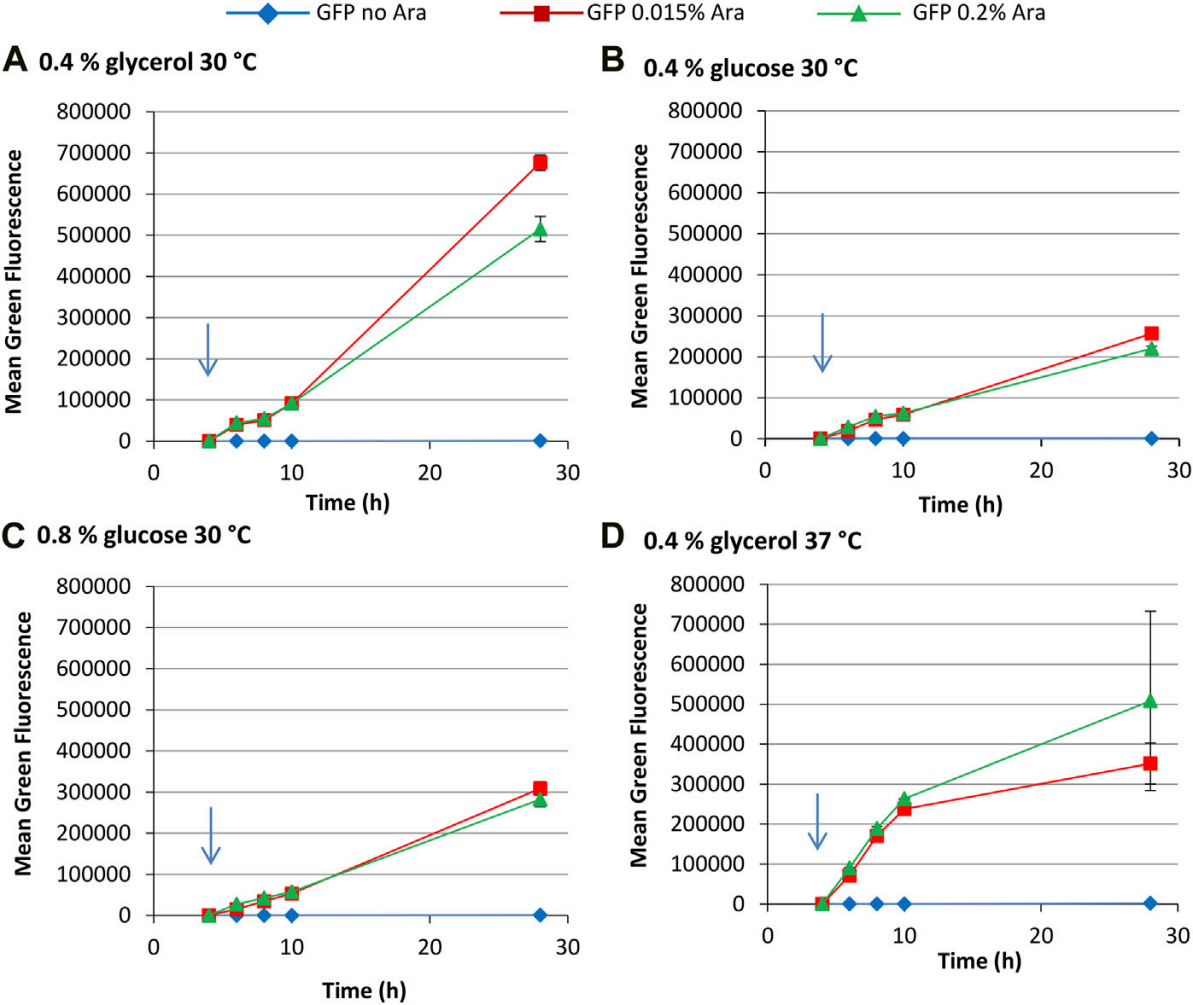
pBAD expression system for the production of GFP. *E. coli* BL21-A cultures carrying pLBAD-GFP was grown at: 30°C in terrific broth with 0.4% glycerol **(A)**; 30°C in terrific broth with 0.4% glucose **(B)**; 30°C in terrific broth with 0.8% glucose **(C)**; or 37°C in terrific broth with 0.4% glycerol **(D)**. At an OD_650_ of 0.5, arabinose (0.015% or 0.2%) was added to four cultures and two were left uninduced. At regular intervals, samples were taken and FCM was used to determine mean green fluorescence. Data points are mean values of duplicate cultures ± SD.

Flow cytometry revealed that there is a more gradual increase in green fluorescence over time compared to the T7 system, with highest fluorescence values reached after 28 h growth (Figure 6). The *araBAD* promoter has far tighter regulation than the T7 system, with uninduced cultures making very little GFP; indeed, tightness of regulation is an advantage of the p*araBAD* system (Guzman et al., 1995). Inspection of single cell fluorescence data reveals that partial induction occurs in the p*araBAD* system in some growth conditions (Supplementary Figure S7). Unlike the T7 system, where partial induction is indicated by a shoulder on the histogram, the *araBAD* promoter appears to have “on” and “off” states. Induction with 0.015% arabinose in the presence of 0.4% glucose results in a non-induced subpopulation 2 h after induction and full induction after a further 2 h. In 0.8% glucose cultures, the non-induced subpopulation is also visible at 4 h post induction. This is expected as glucose represses the *araBAD* promoter (Lichenstein et al., 1987; Guzman et al., 1995). Our data show that glucose represses the p*araBAD* system far more than the T7 system used here. There are very few GFP^−^ cells at the end of growth in cultures grown at 30°C, although more sizable GFP^−^ population at 37°C.

In summary, the T7 system appears best suited to more rapid recombinant protein production, with highest fluorescence values corresponding to high levels of recombinant protein production being reached 4–6 h after induction in the presence of glycerol although growth in the presence of glucose appears to prolong productivity. The T7 system is also far leakier, with uninduced cultures having comparable mean fluorescence to induced cultures after 28 h growth. In contrast, the p*araBAD* system offers more sustained recombinant protein production, tighter control, greater repression by glucose, and a lower impact on the physiology of the cell, leading to far fewer dead cells.

### Bioreactor growth

Following shakeflask growth, cultures expressing GFP and TNFα-GFP using the T7 expression system were intensified in fed-batch bioreactors using a semi-defined medium and glycerol feed (Wyre and Overton, 2014b). Glycerol was chosen as a carbon source rather than glucose as it typically reduces overflow metabolism and thereby acid generation. Cultures were induced with arabinose at the same time as feeding started, at an OD_650_ of around 40 when the initial glycerol in the batch medium was exhausted. As this was a pilot study, a single bioreactor growth experiment was performed for each of GFP and TNFα-GFP.

**FIGURE 7:**
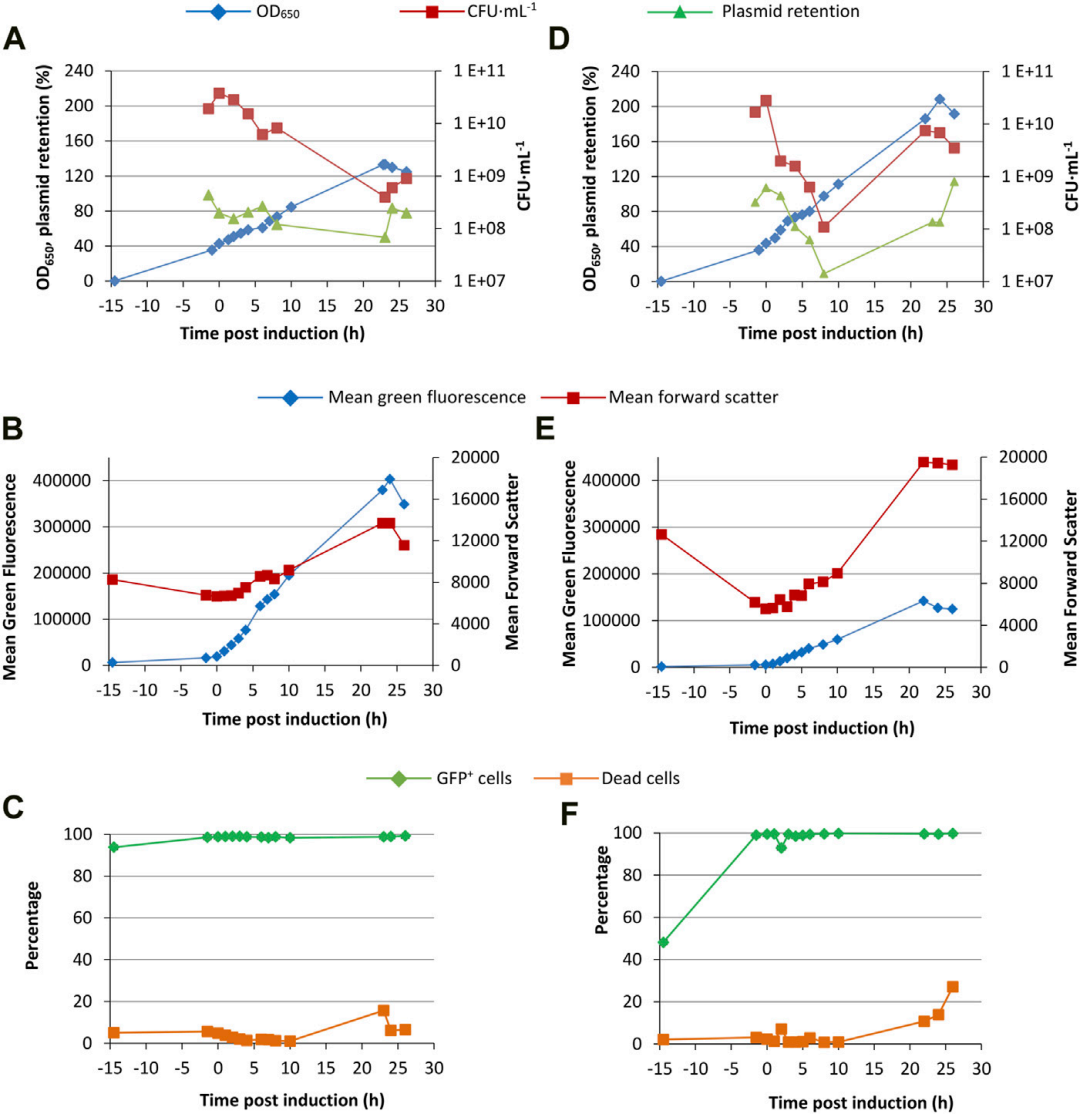
Fed-batch fermentation of the T7 expression system. *E. coli* BL21-T7 cultures carrying either p2T7-GFP [GFP; **(A, B, C)**] or p2T7-TNFaGFP [TNFα-GFP; **(D, E, F)**] were grown at 30°C in a semi-defined medium in a bioreactor as described in the text. Glycerol feed and induction was started as the initial carbon source ran out, as indicated by an increase in DOT. Samples were taken and OD_650_, CFU, and plasmid retention were measured and FCM was used to determine green fluorescence and forward scatter.

The bioreactor culture expressing GFP grew to an OD_650_ of 133 (Figure 7A). The declining CFU·mL^−1^ measurement over time suggests that culture in the bioreactor negatively affected culturability on agar plates; this phenomenon has been observed before and is suggested to be caused by the adaptation of bacteria to the growth conditions within the bioreactor, triggering a viable but non-culturable (VBNC) phenotype (Wyre and Overton, 2014b). FCM analysis (Figure 7C) revealed that the percentage of dead cells remained below 7% except for at 23 h post induction. Online bioreactor measurements indicated a minimum DOT of around 15% (Supplementary Figure S8A). Mean green fluorescence per cell increased steadily following induction (Figure 7B) reaching a maximum of 400 000 units 24 h after induction, and very few cells were not generating GFP (Figure 7C). Green fluorescence histograms showed a single population throughout growth (Supplementary Figure S9A, B), indicative of complete induction, homogeneity of response, and a lack of a plasmid-free GFP^−^ population; the bioreactor culture was far more homogeneous than the shakeflask cultures. The mean forward scatter (an indicator of cell size) increased following induction, as previously observed (Wyre and Overton, 2014b). Overall, the data suggest that the bacteria were productive and physiologically “healthy”, evidenced by a lack of dead or GFP^−^ cells.

Comparing bioreactor and shakeflask growth (Figure 2), maximum mean green fluorescence values are broadly comparable although the bioreactor cultures developed fluorescence far more steadily than the shakeflasks, more akin to the uninduced T7 cultures or the p*araBAD* system. As flow cytometry is a single cell analysis technique, the green fluorescence measured is per cell and therefore the overall yield of the bioreactor would be far higher than shakeflasks due to the much higher biomass concentration; as noted above, multiplication of green fluorescence by biomass concentration would permit direct comparison. Proportions of dead cells were also broadly comparable between the shakeflasks and bioreactor, indicating that the switch to bioreactor growth did not negatively affect cell physiology.

The culture expressing TNFα-GFP attained a higher final OD_650_ (>200; Figure 7D) although mean green fluorescence was lower than the GFP culture (150 000 *versus* 400 000; Figure 7E). As with the GFP culture, green fluorescence histograms showed a single population throughout, with no GFP^−^ population. This suggests that the plasmid loss detected following induction (Figure 7D) was a consequence of the assay requiring growth on agar plates; this is a drawback of such plasmid retention assays, especially with cultures having a VBNC population. The proportion of dead cells was low through the fermentation until around 23 h post induction then rose to 27%, higher than observed in shakeflasks. Overall, these data were broadly similar to TNFα-GFP shakeflask cultures (Figure 2) which had lower mean green fluorescence than GFP cultures, reflecting that GFP is easier to make and fold than TNFα-GFP.

## Conclusion

We have demonstrated that flow cytometry and GFP fusions are a useful approach for process optimisation, with green fluorescence being a rapid readout to determine recombinant protein productivity and solubility. GFP has been used to quantify promoter activity and comparison of GFP and TNFα-GFP cultures has allowed the impact of TNFα on recombinant protein productivity and cell physiology to be determined. Using this approach we have also compared T7 RNA polymerase- and p*araBAD*-based expression systems and found that the latter is more tightly regulated and thereby has less impact on the physiology of *E. coli*. This work could be expanded to study other recombinant protein-GFP fusions; in addition, fluorescence measurement is amenable to high throughput approaches, so expression could be rapidly optimised across a broad experimental space using multiwell plates or multi-vessel bioreactor setups.

## Data Availability

The original contributions presented in the study are included in the article/Supplementary Material, further inquiries can be directed to the corresponding author.
