# Supplementary material for: Optimisation of recombinant TNFα production in Escherichia coli using GFP fusions and flow cytometry
Source: Front Bioeng Biotechnol. 2023 Aug 2;11:1171823. doi: 10.3389/fbioe.2023.1171823 (PMC10433901; doi:10.3389/fbioe.2023.1171823)
Supplement: Supplementary file 1 [file DataSheet1.pdf]

SUPPLEMENTAL INFORMATION

Optimisation of recombinant TNFα production in *Escherichia coli* using GFP fusions and flow cytometry

Nurul Asma Hasliza Zulkifly[1,3], Tania Selas Castiñeiras[1,2], and Tim W Overton[1]*

[1] School of Chemical Engineering and Institute of Microbiology and Infection, The University of Birmingham, Edgbaston, Birmingham, B15 2TT, UK.

[2] Cobra Biologics, Stephenson Building, The Science Park, Keele ST5 5SP, UK.

[3] Current address: School of Agriculture Science and Biotechnology, Faculty of Bioresources and Food Industry, Universiti Sultan Zainal Abidin, Besut Campus, 22200 Besut, Terengganu, Malaysia.

*to whom correspondence should be addressed: t.w.overton@bham.ac.uk +44 (0) 121 414 5306

Supplemental Figure S1. Single cell analysis of T7 expression system cultures at 30 °C with 0.4 % glycerol.

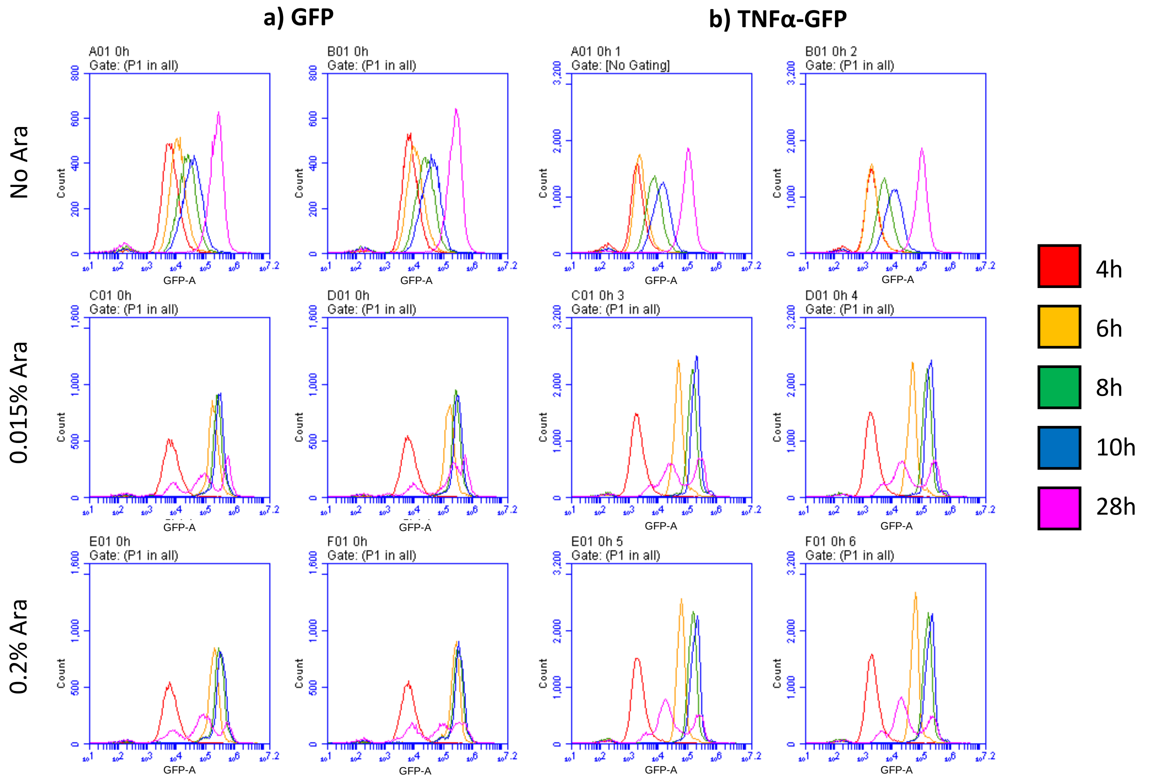

Supplemental Figure S1. Single cell analysis of T7 expression system cultures at 30 °C with 0.4 % glycerol. *E. coli* BL21-T7 cultures carrying either p2T7-TNFaGFP (TNFα-GFP; a) or p2T7-GFP (GFP; b) were grown at 30 °C in terrific broth with 0.4 % glycerol as a carbon source. At an OD_{650} of 0.5, arabinose (0.015% or 0.2%) was added to four cultures and two were left uninduced. At regular intervals, samples were taken and FCM was used to measure green fluorescence of individual cells; left and right plots correspond to duplicate cultures. Timepoints relate to the time the cultures were grown; 4 h refers to pre-induction with arabinose. These data correspond to Figure 1.

Supplemental Figure S2. Single cell analysis of untransformed *E. coli*

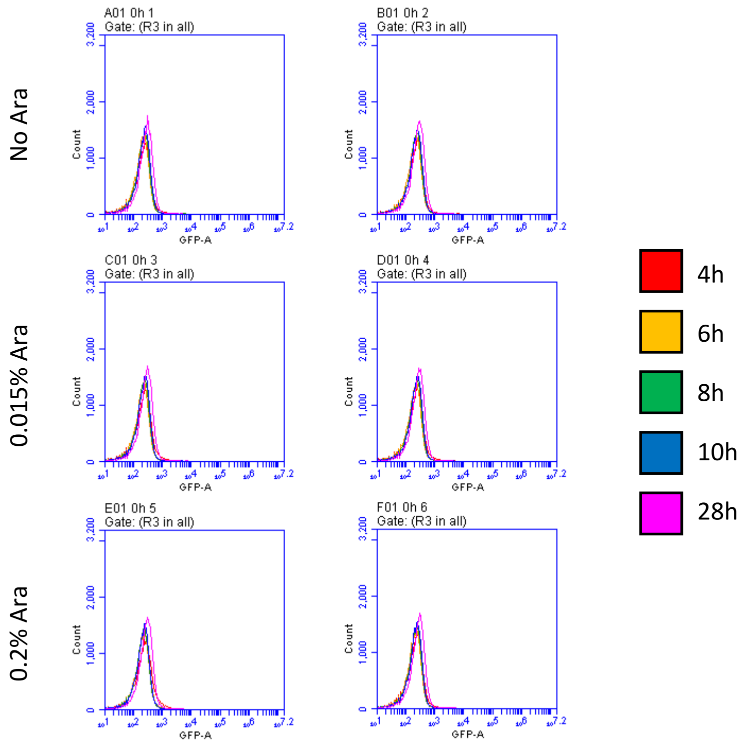

Supplemental Figure S2. Single cell analysis of untransformed *E. coli*. Untransformed *E. coli* BL21-A cultures were grown at 30 °C in terrific broth with 0.4 % glycerol as a carbon source. At an OD_{650} of 0.5, arabinose (0.015% or 0.2%) was added to four cultures and two were left uninduced. At regular intervals, samples were taken and FCM was used to measure green fluorescence of individual cells; left and right plots correspond to duplicate cultures. Timepoints relate to the time the cultures were grown; 4 h refers to pre-induction with arabinose.

Supplemental Figure S3. Single cell analysis of T7 expression system cultures at 30 °C with 0.4 % glucose.

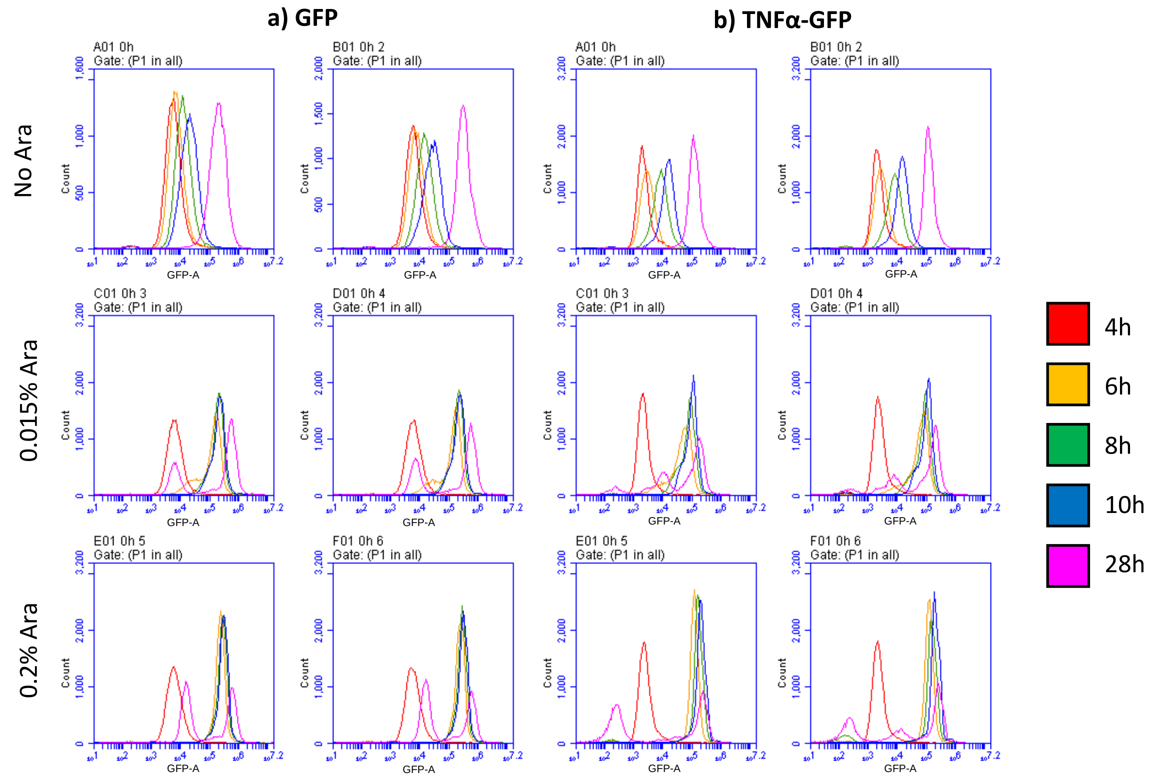

Supplemental Figure S3. Single cell analysis of T7 expression system cultures at 30 °C with 0.4 % glucose. *E. coli* BL21-T7 cultures carrying either p2T7-TNFaGFP (TNFα-GFP; a) or p2T7-GFP (GFP; b) were grown at 30 °C in terrific broth with 0.4 % glucose as a carbon source. At an OD_{650} of 0.5, arabinose (0.015% or 0.2%) was added to four cultures and two were left uninduced. At regular intervals, samples were taken and FCM was used to measure green fluorescence of individual cells; left and right plots correspond to duplicate cultures. Timepoints relate to the time the cultures were grown; 4 h refers to pre-induction with arabinose. These data correspond to Figure 2.

Supplemental Figure S4. Growth and single cell analysis of T7 expression system at 30 °C with 0.8 % glucose.

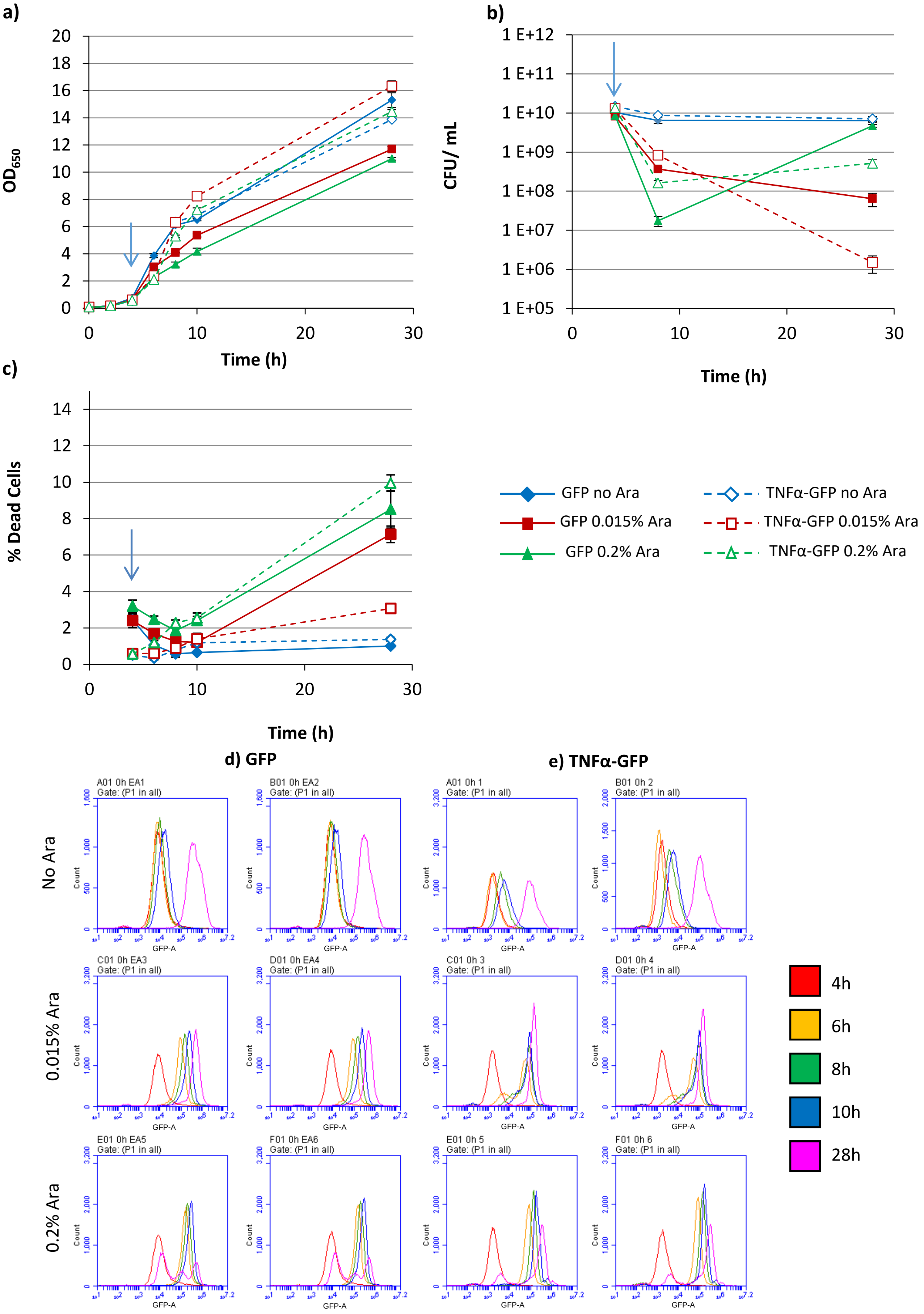

Supplemental Figure S4. Growth and single cell analysis of T7 expression system at 30 °C with 0.8 % glucose. *E. coli* BL21-T7 cultures carrying either p2T7-TNFaGFP (TNFα-GFP) or p2T7-GFP (GFP) were grown at 30 °C in terrific broth with 0.8 % glucose as a carbon source. At an OD_{650} of 0.5 (indicated by an arrow), arabinose (0.015% or 0.2%) was added to four cultures and two were left uninduced. At regular intervals, samples were taken and OD_{650} (a) and CFU (b) were measured. FCM was used to determine the percentage of dead cells (c) after staining with PI. Histograms (d & e) show green fluorescence of individual cells; left and right plots correspond to duplicate cultures. These data correspond to Figure 3.

Supplemental Figure S5. Single cell analysis of T7 expression system cultures at 37 °C with 0.4 % glycerol.

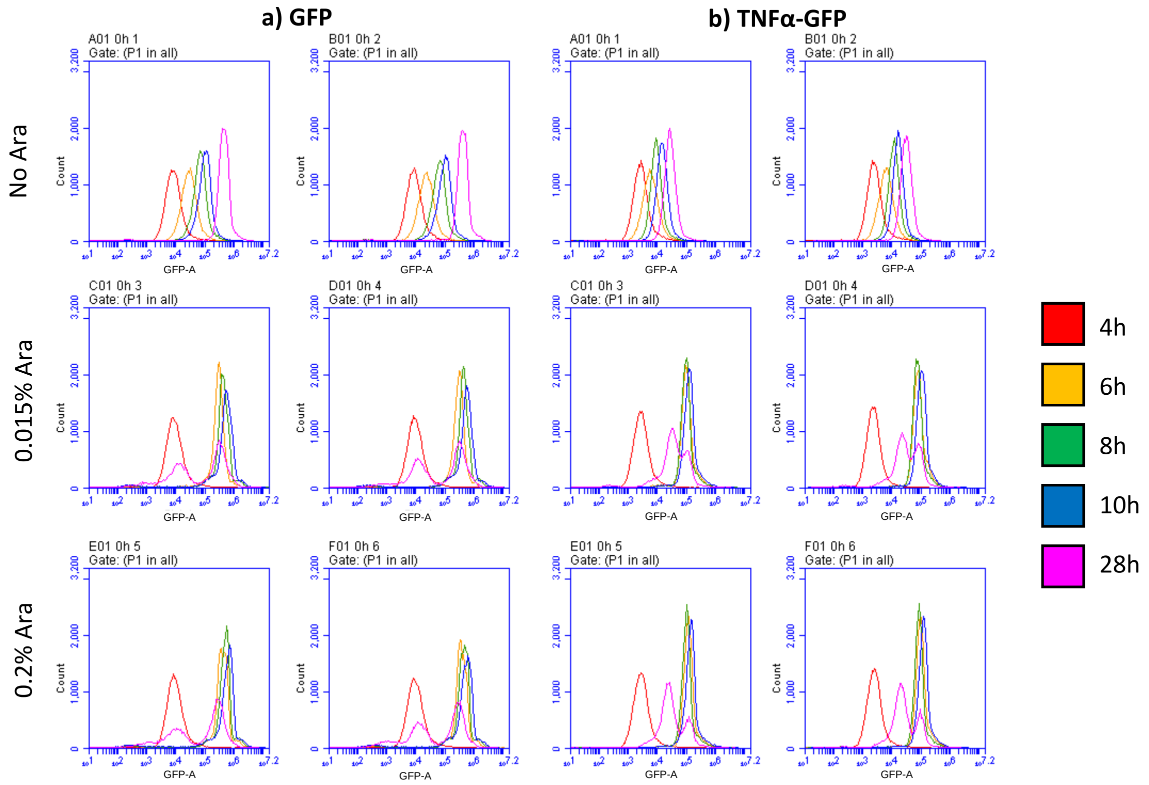

Supplemental Figure S5. Single cell analysis of T7 expression system cultures at 37 °C with 0.4 % glycerol. *E. coli* BL21-T7 cultures carrying either p2T7-TNFaGFP (TNFα-GFP; a) or p2T7-GFP (GFP; b) were grown at 37 °C in terrific broth with 0.4 % glycerol as a carbon source. At an OD_{650} of 0.5, arabinose (0.015% or 0.2%) was added to four cultures and two were left uninduced. At regular intervals, samples were taken and FCM was used to measure green fluorescence of individual cells; left and right plots correspond to duplicate cultures. Timepoints relate to the time the cultures were grown; 4 h refers to pre-induction with arabinose. These data correspond to Figure 4.

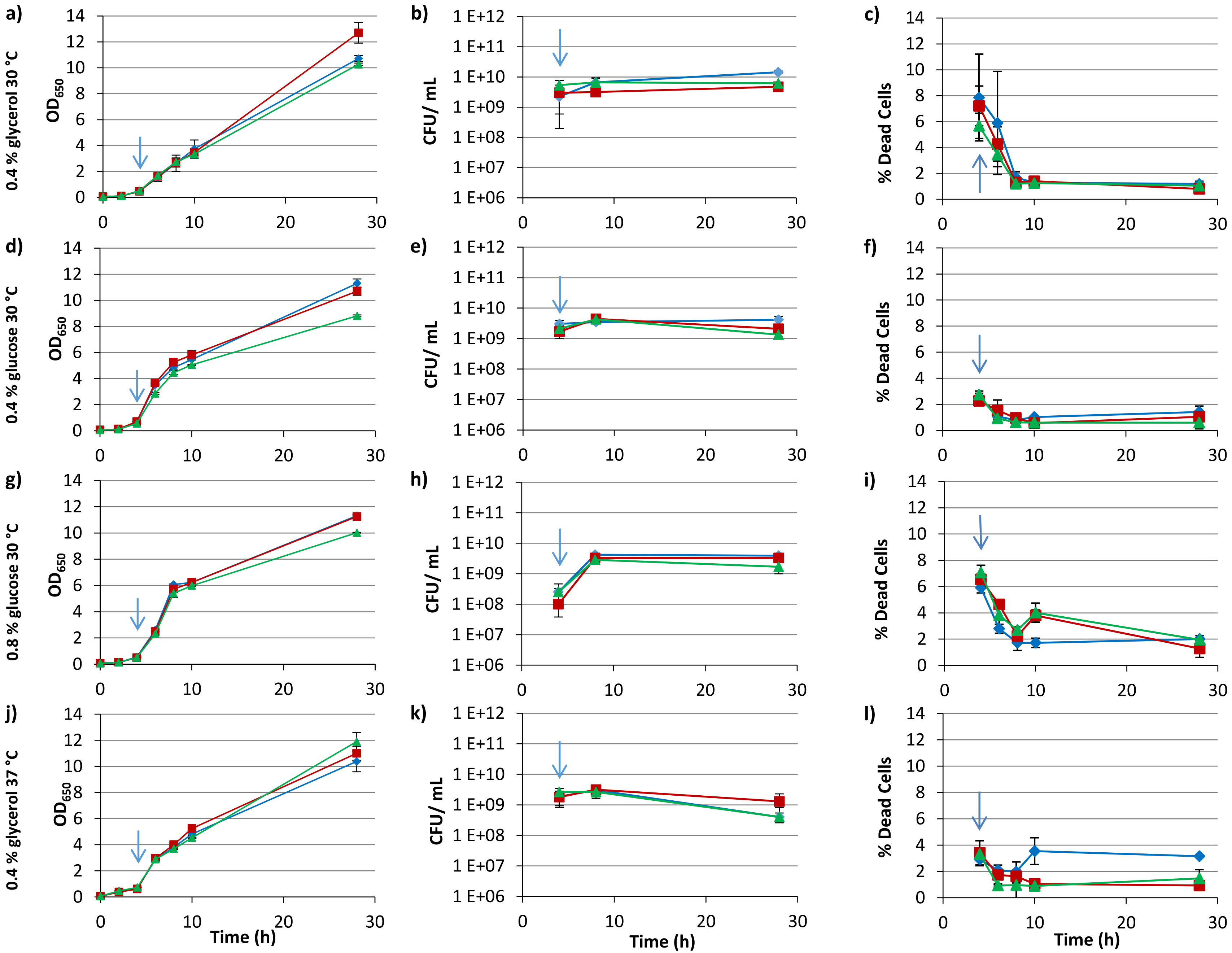

a)
b)
c)
d)
e)
f)
g)
h)
i)
j)
k)
l)
0.4 % glycerol 30 °C
0.4 % glucose 30 °C
0.8 % glucose 30 °C
0.4 % glycerol 37 °C
OD650
CFU/ mL
% Dead Cells
Time (h)

Supplemental Figure S6. pBAD expression system for the production of GFP. *E. coli* BL21-A cultures carrying pLBAD-GFP was grown at: 30 °C in terrific broth with 0.4 % glycerol (a-c); 30 °C in terrific broth with 0.4 % glucose (d-f); 30 °C in terrific broth with 0.8 % glucose (g-i); or 37 °C in terrific broth with 0.4 % glycerol (j-l). At an OD_{650} of 0.5 (indicated by an arrow), arabinose (0.015% or 0.2%) was added to four cultures and two were left uninduced. At regular intervals, samples were taken and OD_{650} (a,d,g,j) and CFU (b,e,h,k) were measured and FCM was used to determine the percentage of dead cells following staining with PI (c,f,i,l).

Supplemental Figure S7. Single cell analysis of pBAD expression system cultures.

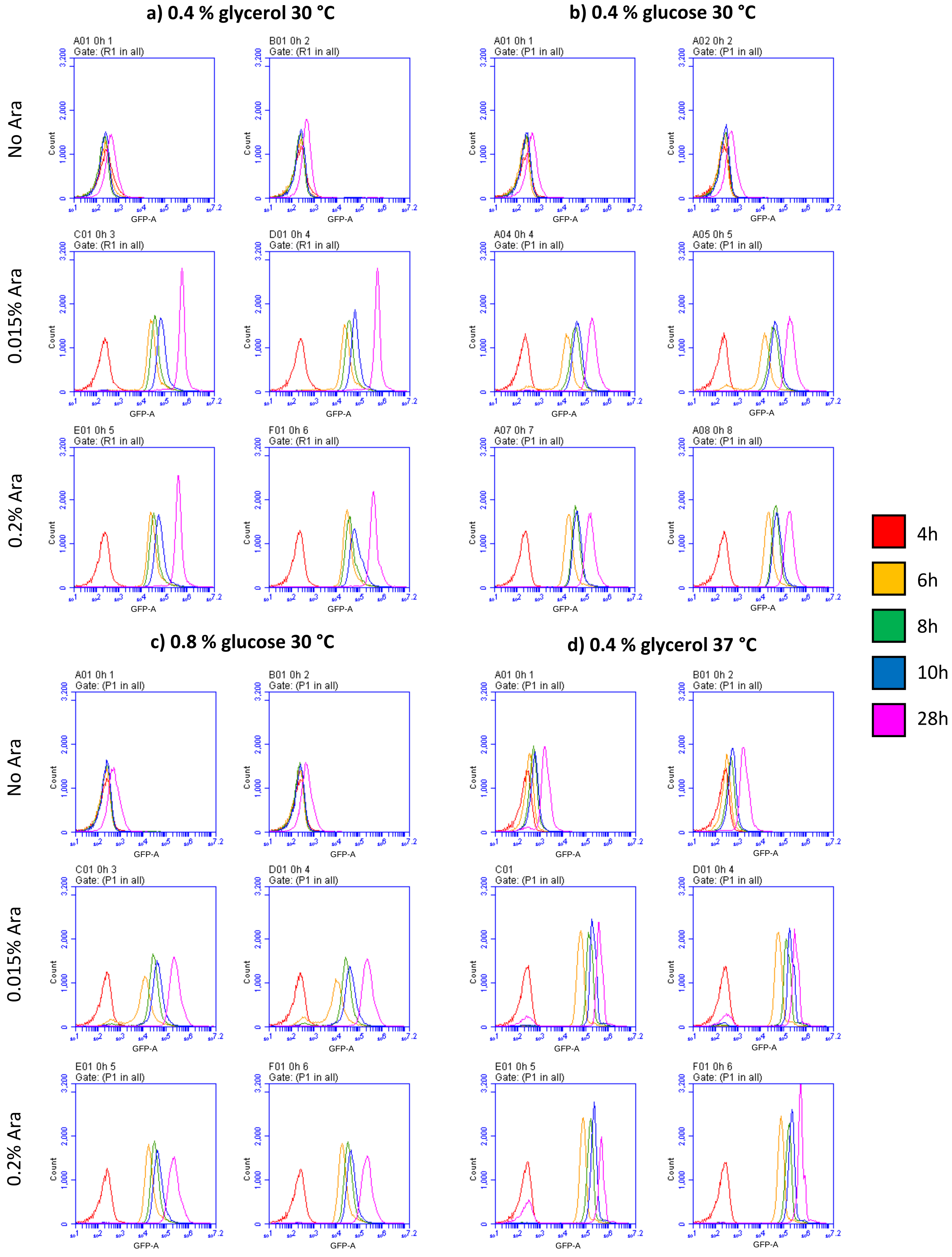

Supplemental Figure S7. Single cell analysis of pBAD expression system cultures. *E. coli* BL21-A cultures carrying pLBAD-GFP was grown at: 30 °C in terrific broth with 0.4 % glycerol (a); 30 °C in terrific broth with 0.4 % glucose (b); 30 °C in terrific broth with 0.8 % glucose (c); or 37 °C in terrific broth with 0.4 % glycerol (d). At an OD_{650} of 0.5, arabinose (0.015% or 0.2%) was added to four cultures and two were left uninduced. At regular intervals, samples were taken and FCM was used to measure green fluorescence of individual cells; left and right plots correspond to duplicate cultures. Timepoints relate to the time the cultures were grown; 4 h refers to pre-induction with arabinose. These data correspond to Figure 5 and Supplemental Figure S6.

Supplemental Figure S8. Fermentation data for T7 expression system cultures in bioreactors.

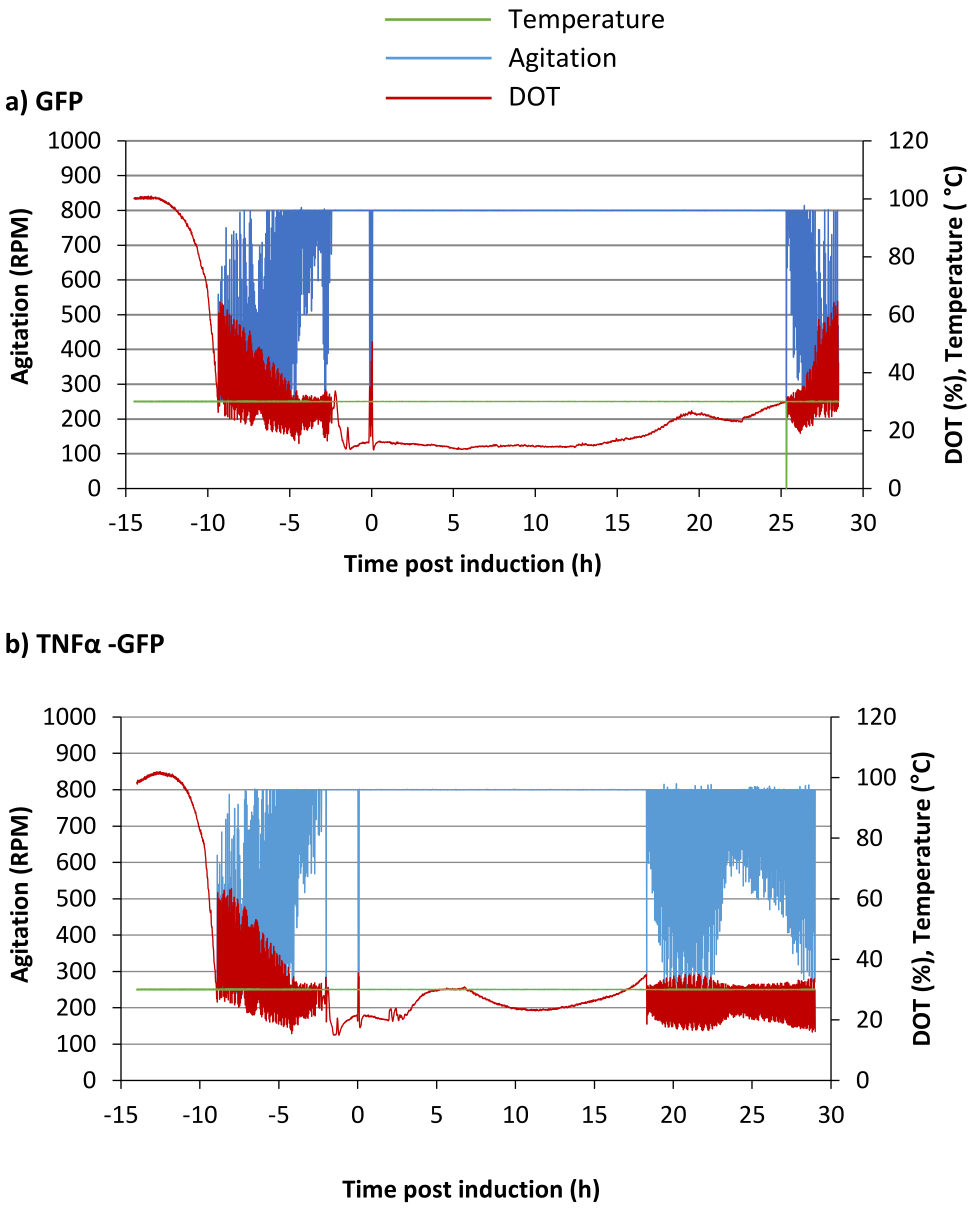

Supplemental Figure S8. Fermentation data for T7 expression system cultures in bioreactors. *E. coli* BL21-T7 cultures carrying either p2T7-GFP (GFP; a) or p2T7-TNFaGFP (TNFα-GFP; b) were grown at 30 °C in a semi-defined medium in a bioreactor as described in the text. Temperature, DOT and agitation speed were continuously logged by the bioreactor.

Supplemental Figure S9. Single cell analysis of T7 expression system cultures in bioreactors

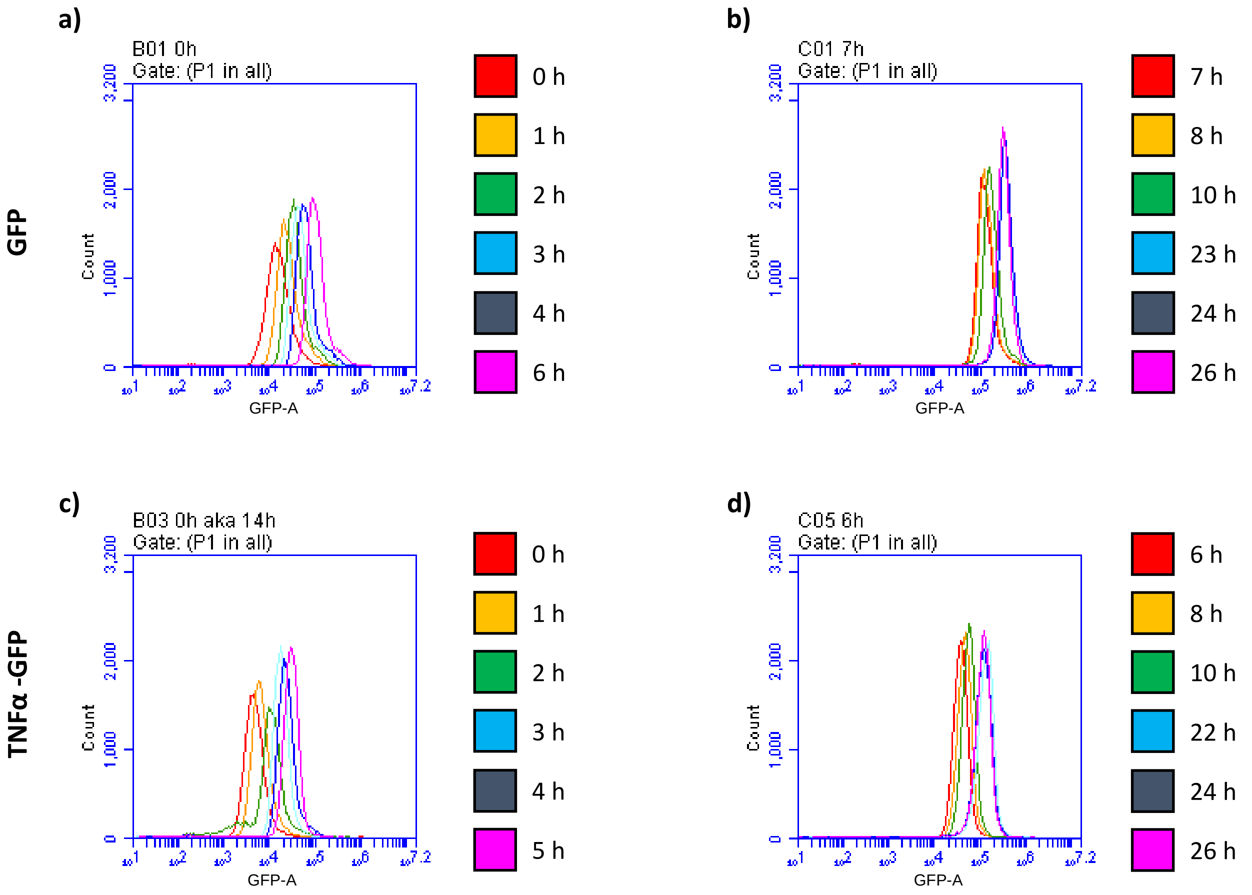

Supplemental Figure S9. Single cell analysis of T7 expression system cultures in bioreactors. *E. coli* BL21-T7 cultures carrying either p2T7-GFP (GFP; a,b) or p2T7-TNFaGFP (TNFα-GFP; c,d) were grown at 30 °C in a semi-defined medium in a bioreactor as described in the text. Samples were taken at regular intervals and analysed by FCM. Histograms show green fluorescence of individual cells. These data correspond to Figure 6 and Supplemental Figure S8.
